# Plant Viruses: From Targets to Tools for CRISPR

**DOI:** 10.3390/v13010141

**Published:** 2021-01-19

**Authors:** Carla M. R. Varanda, Maria do Rosário Félix, Maria Doroteia Campos, Mariana Patanita, Patrick Materatski

**Affiliations:** 1 MED—Mediterranean Institute for Agriculture, Environment and Development, Instituto de Investigação e Formação Avançada, Universidade de Évora, Pólo da Mitra, Ap. 94, 7006-554 Évora, Portugal; mdcc@uevora.pt (M.D.C.); mpatanita@uevora.pt (M.P.); 2 MED—Mediterranean Institute for Agriculture, Environment and Development & Departamento de Fitotecnia, Escola de Ciências e Tecnologia, Universidade de Évora, Pólo da Mitra, Ap. 94, 7006-554 Évora, Portugal; mrff@uevora.pt

**Keywords:** CRISPR/Cas systems, viral vectors, gene editing, plant genome engineering, viral resistance

## Abstract

Plant viruses cause devastating diseases in many agriculture systems, being a serious threat for the provision of adequate nourishment to a continuous growing population. At the present, there are no chemical products that directly target the viruses, and their control rely mainly on preventive sanitary measures to reduce viral infections that, although important, have proved to be far from enough. The current most effective and sustainable solution is the use of virus-resistant varieties, but which require too much work and time to obtain. In the recent years, the versatile gene editing technology known as CRISPR/Cas has simplified the engineering of crops and has successfully been used for the development of viral resistant plants. CRISPR stands for ‘clustered regularly interspaced short palindromic repeats’ and CRISPR-associated (Cas) proteins, and is based on a natural adaptive immune system that most archaeal and some bacterial species present to defend themselves against invading bacteriophages. Plant viral resistance using CRISPR/Cas technology can been achieved either through manipulation of plant genome (plant-mediated resistance), by mutating host factors required for viral infection; or through manipulation of virus genome (virus-mediated resistance), for which CRISPR/Cas systems must specifically target and cleave viral DNA or RNA. Viruses present an efficient machinery and comprehensive genome structure and, in a different, beneficial perspective, they have been used as biotechnological tools in several areas such as medicine, materials industry, and agriculture with several purposes. Due to all this potential, it is not surprising that viruses have also been used as vectors for CRISPR technology; namely, to deliver CRISPR components into plants, a crucial step for the success of CRISPR technology. Here we discuss the basic principles of CRISPR/Cas technology, with a special focus on the advances of CRISPR/Cas to engineer plant resistance against DNA and RNA viruses. We also describe several strategies for the delivery of these systems into plant cells, focusing on the advantages and disadvantages of the use of plant viruses as vectors. We conclude by discussing some of the constrains faced by the application of CRISPR/Cas technology in agriculture and future prospects.

## 1. Introduction

Plant viruses are known to infect and cause devastating diseases in many agricultural systems, leading to significant losses in crop quality and yield, with extreme economic impacts worldwide, being a serious threat for the provision of adequate nourishment to a continuous growing population [1,2]. Climate change has been rapidly causing aggravation of viral disease impacts, with existing virus showing pandemic behavior, and with the appearance of new emergent viruses, making the development of efficient long term disease management approaches difficult [3].

Plant viruses are obligate intracellular pathogens and at present there are no chemical products that directly target the virus, that can be used in agronomic context, making preventive sanitary measures the only way to hamper infections. Preventive sanitary measures consist mostly of good sanitation techniques during cultural practices, that include the immediate removal and destruction of infected plants, the limitation of the virus vector organisms populations and the development of legislative measures concerning the commercialization and trade of virus free plant material [4]. Many of these conventional strategies are unsafe for the environment and have proved to be far from enough. The use of viral resistant plants is currently the most efficient and sustainable solution to reduce viral infections. Thus, it is essential to develop effective and durable virus resistant varieties to face the increasingly severe viral diseases and viral variants [5,6,7,8]. For many years, classical breeding for crop improvement involved the selection of plants with certain agronomic characteristics and absence of viral symptoms, a very laborious and time-consuming strategy [9].

Advances in biotechnology have provided new knowledge on molecular mechanisms of plant virus interactions, which accelerated the process of breeding through approaches based on molecular marker-assisted breeding, genomic selection, gene silencing, pathogen-derived resistance (PDR), etc., and has provided many resistant varieties to agriculture [10,11,12]. However, the rapid evolution and emergence of new viruses makes the durability of the resistance a major drawback and creates the need of rapid and efficient techniques for obtaining resistant plants.

In recent years, the versatile gene editing technology known as CRISPR/Cas has simplified the engineering of crops and has already been used for the development of resistance to viral pathogens, overcoming many difficulties of the techniques used to date [13,14,15].

Moreover, viruses can be manipulated to be beneficial and useful for several purposes as they present an efficient machinery and a comprehensive genome structure. They have been used in biotechnology as molecular tools in several areas such as medicine, materials industry, and agriculture with different purposes including the production of proteins and being targets and vectors of many materials [16,17]. Due to all this potential, it is not surprising that viruses have also been used in this revolutionary genome editing technique.

In this review, we start by describing the basic principles of CRISPR/Cas technology, with special focus on the advances of CRISPR/Cas to engineer plant resistance against RNA and DNA viruses. We demonstrate that, for the successful use of this technology, it is imperative that the CRISPR/Cas system is efficiently delivered and expressed in the targeted cells, and we describe several strategies for the delivery of these systems into plant cells. In a different perspective, we show how viruses can be manipulated to be used as tools for the delivery of CRISPR/Cas systems into plant cells, focusing on the advantages and disadvantages of the use of viruses as vectors of CRISPR systems into plant cells. We conclude by discussing the constrains faced by CRISPR/Cas technology and the future prospects.

## 2. CRISPR: From a Natural Bacterial Immune System to a Gene Editing Tool

Clustered regularly interspaced short palindromic repeats (CRISPR) and CRISPR-associated (Cas) proteins is a natural adaptive immune system that some bacterial and most archaeal species present to defend themselves against invading bacteriophages, which works on the basis of sequence complementarity via cleavage [18,19].

CRISPR systems may be divided into two main classes (I and II) and six different types (I to VI), defined by the nature of the nucleases complex and the mechanism of targeting, each presenting a unique nuclease Cas protein. Class I systems are multicomponent systems composed of multiple effectors; these systems are subdivided into types I, III, and IV. Class II systems include the types II, V, and VI and are single-component systems consisting of a single effector guided by the CRISPR RNA (crRNA) [20].

The CRISPR/Cas9, belonging to class II, is based on the immune system of *Streptococcus pyogenes*. It consists of the capacity of the bacteria to acquire pieces of DNA from an invading phage or plasmid and incorporating them in their own DNA, which will further serve to guide Cas9 to cleave homologous RNA, leading to immediate RNA disruption and further specific RNA disruption in subsequent invasions, thus providing immunity to the bacterial cell [21]. The mechanism involved in this natural immune system is very simple and has been the basis for the most developed CRISPR/Cas genome-editing platform.

The first steps of CRISPR/Cas9 as a successful editing tool, started with the possibility of engineering into a single RNA chimera (sgRNA), two noncoding RNAs essential for CRISPR, crRNA, and trans-activating crRNA (tracrRNA) [22]. crRNA is the genomic complementary region, i.e., the target for Cas (the programmable portion defined by the user) and tracrRNA is the RNA sequence that provides the stem loop structure to bound Cas. This has simplified gene editing using CRISPR/Cas9, which can now be accomplished by introducing two components in the same cell: the sgRNA and the Cas protein [22] and led to efficient genetic manipulation in a wide array of plants, becoming the most promising, versatile, and powerful tool for plant improvement [23].

**Figure 1:**
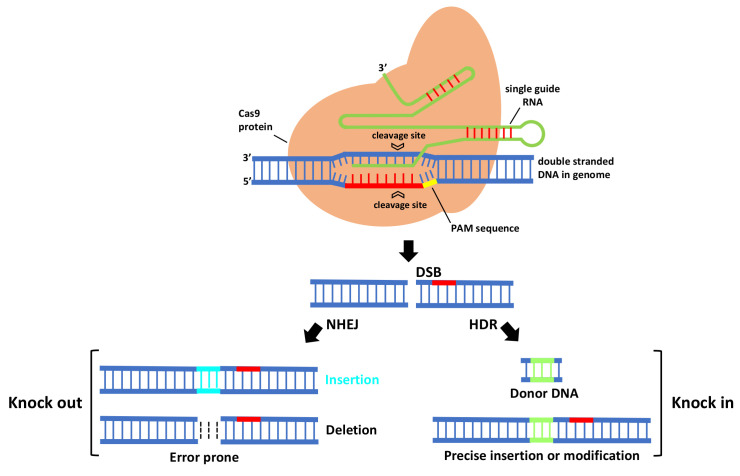
The mechanism of CRISPR-Cas9-mediated genome engineering in plants. A single guide RNA recognizes a region in the genome followed by a PAM sequence, and recruits a Cas9 protein that will cleave DNA, creating a double-stranded break that is repaired by error-prone non-homologous end-joining (NHEJ) and homology-directed repair (HDR).

In CRISPR/Cas9 system (Figure 1), first Cas9 binds to the sgRNA to create the Cas9-sgRNA duplex which becomes catalytically active and directs the RNA-guided DNA endonuclease Cas9 to target. For target recognition and cleavage, it is also required the presence of a Protospacer Adjacent Motif (PAM) positioned 3–4 nucleotides downstream of the 3′ end of the target sequence, which differs depending on the species of Cas9 (this sequence consists of NGG in *S. pyogenes*) [22,24]. Once the PAM sequence is recognized by the Cas9-sgRNA complex, and the crRNA portion within the sgRNA (the 5′ most 20 nts) anneals to the genomic DNA through Watson–Crick base pairing, it will cleave both DNA strands, three bases upstream of the PAM, creating sequence-specific blunt end double-stranded breaks (DSBs) at target site. When a DSB in the DNA is created, the host cell repairs it via evolutionary conserved DNA pathways such as error-prone non-homologous end-joining (NHEJ) and homology-directed repair (HDR).

NHEJ creates insertions or deletions (indels) at the target site that, if within the protein coding region, can cause a frameshift mutation that eliminates gene expression, leading to gene knock out [25]. HDR is a more precise method for DSB repair; it requires, besides sgRNA and Cas, a donor repair template with ends homologous to each border of the target site sequence. When a repair template is provided, HDR will result in the introduction of new sequences at breaking site and a knock in occurs [25]. For producing specific desired mutations and genomic replacement, DSBs should be repaired by HDR pathway. More recently, a new generation of CRISPR is being developed by fusing nuclease DNA targeting proteins with deactivated nuclease domains, with enzymes to enable direct conversion of a single DNA nucleotide into another [26] without the need of DSB formation.

Genetic engineering using CRISPR/Cas systems enables accurate and precise genomic modifications. Moreover, this strategy can be used to target different sequences simultaneously with high efficiency [27], achieving a broader result, as for example immunity against different pathogens.

The easiness and rapidity of execution, low cost, reproducibility and efficiency turns understandable why it is the system of choice for many genome engineering applications in several fields using different organisms. The possibility of using Cas proteins with deactivated nuclease domains can contribute to a broader application of CRISPR such as regulating gene transcription and inducing targeted epigenic modifications [28]. In addition, CRISPR has shown to have potential for other applications besides genome engineering, such as studies on gene functions and diagnostics. CRISPR/LwaCas13a system was able to highly select and detect up to a single copy of RNA [29], which may be a very interesting starting point to develop a far more sensitive method than currently available methods, for the detection of RNA viruses, including qPCR [30].

In plants, this technology has been used for plant breeding including nutrition enhancement and plant resistance against several agents such as fungi, bacteria, and viruses in many crop plants—including rice [31], tomato [32], citrus [33,34], wheat [35], and maize [36,37]—proving its potential to transform agriculture and enhancing world food safety.

## 3. CRISPR to Engineer Plant Virus Resistance

Due to the devastating losses that plant viruses cause, it is not surprising that CRISPR/Cas technologies have been applied to develop plant resistance against viral pathogens.

Plant viral resistance using CRISPR/Cas systems can been achieved either through manipulation of plant genome (plant-mediated resistance), or virus genome (virus-mediated resistance).

The CRISPR/Cas technology was initially thought to be exclusively applied to DNA, which, in terms of its use for plant viral resistance through manipulation of viral genome, would be restricted to DNA viruses. However, thanks to the discovery of RNA-targeting CRISPR/Cas effectors that efficiently target and cleave single-stranded RNAs, an exciting opportunity has been opened for achieving plant resistance also against RNA viruses, which are most of the plant viruses known [38,39].

Below we present several studies that report the use of the CRISPR/Cas system to engineer plant resistance against several viruses, either by acting on plant genome (plant mediated resistance) or on viral genomes (virus mediated resistance). These studies have shown the capacity of CRISPR to confer efficient and durable molecular immunity to plants against viruses that rely on the integrity of their genome at some point of their replication cycle [15,40,41,42,43].

### 3.1. CRISPR for Plant Mediated Resistance

Plant viruses are dependent on the host’s machinery for their replication, since they interact with many host factors required for viral replication and movement inside plants, essential to complete their cycle of infection [44]. CRISPR/Cas allows the mutation/deletion of recessive genes that encode critical host factors for viral infection, conferring recessive resistance, which, as an inherited characteristic is very durable [45].

Considerable knowledge has been generated on the genetics of plant disease resistance and many plant genes have been discovered as essential for viral infections and have been the focus for the development of plant resistance using transgenic approaches [12,46,47]. These studies have provided many valuable potential targets for genome editing and genes—such as the translation initiation-like factors elF4E, elF4G, and their isoforms—that have shown to be directly involved in the infection process of viruses. Those genes are being subjected to targeted mutations introduced by CRISPR to engineer plant resistance [48]. In fact, any host gene encoding a factor required by the virus is a potential target for CRISPR.

This approach is interesting as it allows that Cas9, as well as other endonucleases which target DNA, to be used to provide plant resistance to RNA viruses by mutating host factors/genes associated to viral pathogenesis in the plant [49]. In addition, CRISPR for plant mediated resistance does not require the maintenance of a transgene for Cas9 and sgRNA in the plant genome, engineering transgenic-free virus-resistant plants [14,42,49].

Several studies have achieved plant mediated resistance against viruses using CRISPR/Cas9 (Table 1). For example, specific mutations were introduced in *Arabidopsis thaliana*, causing the knock out of elF(iso)4E gene, which resulted in a stable resistance against *Turnip mosaic virus* (TuMV) [42]. Macovei et al. [50] developed rice plants resistant to *Rice tungro spherical virus* (RTSV) through mutation of elF4G gene. Similarly, the disruption of the cucumber (*Cucumis sativus*) elF4E gene provided plant resistance to multiple members of the *Potyviridae*, namely the ipomovirus *Cucumber vein yellowing virus* (CVYV) and the potyviruses *Zucchini yellow mosaic virus* (ZYMV) and *Papaya ringspot mosaic virus* (PRSV) [49]. Resistance against *Clover yellow vein virus* (CYVV) was achieved in *A. thaliana* plants by targeting the elF4E1 gene using CRISPR/Cas9 [51]. Very recently, CRISPR/Cas9 has also allowed to perform double mutations on the novel cap-binding protein-1 and protein-2 (nCBP-1 and nCBP-2) belonging to the elF4E family, on cassava, which increased the resistance to *Cassava brown streak virus* (CBSV) [52].

It is a fact that modifications of plant genes may always face the risk to interfere with plant functions associated to those genes, with a fitness cost for the host, however these examples have demonstrated the success of CRISPR/Cas9 to produce genetic resistant plants through plant mediated resistance and without compromising plant functions.

### 3.2. CRISPR for Virus Mediated Resistance

Another approach to achieve plant viral resistance through CRISPR systems is by directly targeting viral genomes. In this approach, the problems that may arise by interfering with genes, that may also be associated to other plant functions—such as growth, reproduction, or others—are surpassed. However, for this type of mediated resistance, CRISPR/Cas systems must specifically directly target and cleave DNA of DNA viruses, or RNA of RNA viruses [43].

CRISPR for virus mediated resistance was first exploited to fight DNA viruses, as the discovery of CRISPR/Cas systems that can cleave RNA was more recent [27,39]. The discovery of such systems (class II, type VI Cas effectors, and Cas9 variants)—namely Cas13a (C2c2), Cas13b (C2c6), Cas13c (C2c7), Cas13d, FnCas9, and RCas9 (RNA targeting SpCas9) [20,27,53,54,55,56], was a great benefit—enabling direct targeting of RNA viruses which represent most plant pathogenic viruses.

Several studies have demonstrated the potential of CRISPR to impart plant resistance by targeting either DNA or RNA viral genomes, causing delayed or reduced accumulation of viruses and significantly attenuating symptoms of infection [57]. Some of those studies which directly mutate DNA and RNA viruses in plants expressing CRISPR/Cas machinery are described below (Table 1).

There are two major groups of plant DNA viruses, the double stranded caulimoviruses and the geminiviruses, the later which, although single stranded, replicate within the plant cell as double stranded DNA [58]. According to the latest report of the international Committee on Taxonomy of Viruses (ICTV), the *Geminiviridae* is the largest group of plant viruses, with 485 species [59]. Geminiviruses infect many economically important crops such as cassava, watermelon, squash, petunia, tobacco, pepper, potato, tomato, bean, soybean, cowpea, cotton, and others, leading to reduced crop yields worldwide [60,61]. Due to this reason, it is not surprising that most DNA virus mediated resistance studies have been applied to geminiviruses (Table 1). Ali et al. [62] used sgRNA molecules targeting coding (rep genes and coat proteins) and non-coding sequences (conserved intergenic region) of the *Tomato yellow leaf curl virus* (TYLCV) genome, that were delivered via *Tobacco rattle virus* (TRV) system into *Nicotiana benthamiana* plants expressing Cas9, causing a reduction of accumulation of viral DNA and reduction of symptoms in plants. A subsequent study using CRISPR/Cas9 system with a sgRNA targeting a conserved region in multiple begomoviruses (CLCuKoV, TYLCV, TYLCSV, MeMV, BCTV-Worland and BCTV-Logan), simultaneously mediated interference and showed that the targeting of viral non-coding, intergenic sequences was more efficient, limiting the generation of recovered viral variants that evade CRISPR-mediated immunity by reverting the induced mutations through NHEJ [40]. Other studies have achieved plant viral resistance through the expression of sgRNAs complementary to sequences either within *Bean yellow dwarf virus* (BeYDV), *Wheat dwarf virus* (WDV) or *Beet severe curly top virus* (BSCTV) genomes, which reduced virus accumulation and symptoms in plants overexpressing Cas9 such as *N. benthamiana*, barley, and *A. thaliana* [41,63,64]. Similarly, CRISPR/Cas9 allowed to obtain resistance against banana streak disease by targeting endogenous *Banana streak virus* (eBSV) sequences [65].

Plant resistance to a caulimovirus was achieved when Liu et al. [38] expressed multiple sgRNAs targeting the caulimovirus *Cauliflower mosaic virus* (CaMV) coat protein gene in Arabidopsis plants and 20 days after mechanical inoculation of the virus, 85–90% of the plants remained symptomless and showed no presence of CaMV.

Immunity against the RNA viruses *Cucumber mosaic virus* (CMV) and *Tobacco mosaic virus* (TMV) was achieved in *N. benthamiana* and *A. thaliana* transgenic plants expressing FnCas9 and a sgRNA complementary to viral genome delivered through a pCambia based vector [13]. Another study showed that *N. benthamiana* expressing Cas13a either transiently (using binary vector pK2WG_7_) or constitutively, and expressing crRNAs complementary to different *Tulip mosaic virus* (TuMV) genomic regions, delivered through TRV system, interfered with viral replication and spread [39]. CRISPR/Cas13a (LshCas13a) system showed to target and degrade genomic RNA of TMV in *N. benthamiana* plants and to confer resistance to *Southern rice black-streaked dwarf virus* (SRBSDV) and *Rice stripe mosaic virus* (RSMV) in rice plants [15]. Zhan et al. [66] showed that transgenic potato lines expressing Cas13a/sgRNA constructs targeting conserved coding regions of different *Potato virus Y* (PVY) strains allowed to confer broad spectrum resistance against multiple PVY strains.

As stated above, many studies have shown the great versatility of the CRISPR technology towards plant virus resistance and have successfully shown the production of viral resistant plants. CRISPR has the potential to accelerate viral resistance breeding, since it is more effective and rapid than conventional breeding. In addition, CRISPR has the capacity to target virus directly and therefore to be applied to crops with limited genome sequence information.

There are also limitations of the use of CRISPR in virus plant resistance that must not be discarded. Knocking out essential host factors may always lead to the possibility of plant lethality or impaired growth [67,68]. Although many studies concerning mutations of host factors did not report any negative effects, the introduction of point mutations in host factor genes, instead of knocking out, should be considered, so that it does not interfere with plant growth but still prevents viral infection [69]. Another important limitation of CRISPR is the undesirable genomic modifications of plant genome, the off-targets. Although much less common to occur in plants than in other systems, off-target mutations may be avoided by the use of catalytically inactive Cas nucleases [70] or by using systems that only target RNA, which will be further destroyed by the plant silencing system.

CRISPR/Cas requires the optimal selection of sgRNA target sites to ensure that targeted viruses do not evolve mutations that escape from CRISPR/Cas cleavage, and that novel and more severe strains that cannot be cleaved again do not arise [40,71]. Additionally, multiplex targeting and targeting noncoding regions of viral genomes have shown to reduce viral mutation rates and minimize the formation of new viral strains capable of infection [40]. Also, CRISPR/Cas systems that target or bind RNA can be used together with Cas9 to reduce the RNA intermediates of DNA viruses, eliminating the viruses that may escape the CRISPR/Cas9 machinery [40]. FnCas9 has shown binding capacity to viral transcripts which probably provides even more durable resistance than nucleases that provide direct targeting [43].

There is still a long way to go concerning the full potential of CRISPR/Cas systems for engineering plant virus resistance, and more studies still need to be performed to improve their efficiency. However, it is clear that CRISPR is a milestone in plant virus resistance and the utilization of this technology in agriculture will certainly result in higher yields and quality of plants.

## 4. Delivery and Expression of CRISPR Systems in Plants

One crucial step in CRISPR for achieving a highly efficient genome engineering technology is the delivery and expression of CRISPR/Cas components within a plant cell [72], which greatly influences the editing efficiency.

If alien DNA is introduced in the host in a way that it gets incorporated into host genome (transgenic plants), a stable expression is provided and higher editing efficiencies may be obtained, but it is more likely that undesirable off-target mutations are originated [73]. On the other hand, if introduced DNA does not get incorporated into host genome and is expressed transiently, the host is considered free from the alien DNA or simply DNA-free.

Transient expression may be achieved by using ribonucleoproteins (RNP) or plasmids or other vectors delivered by agroinfiltration, carrying CRISPR/Cas components. Several studies have used CRISPR by expressing both Cas and sgRNA constitutively, both transiently or either Cas or sgRNA transiently and the other constitutively [72].

Transient expression of Cas endonuclease reduces off-target modifications, while maintaining a high expression of the sgRNAs that would be constitutively being expressed in the plant. However, this situation involves the use of two different plasmids (which would increase to three if a donor DNA was used for knock in). Transient expression of all CRISPR/Cas components (if no donor for DNA repair is used) can obtain DNA-free plants, avoiding the hurdles associated to transgenic plants.

Either way, it is desirable that CRISPR/Cas components are expressed in germline cells, which easily occurs in stable integration, as all cells in transgenic plants will express the CRISPR system, but which may not occur in transient expression. In this case, CRISPR/Cas components must be introduced directly into germline cells or be able to migrate to these cells, thus allowing mutations to be transmitted to the next generation of plants, without the need of tissue culture and all the labor and time consumption it implies.

Several methods have been used to introduce CRISPR/Cas components in plants, including Agrobacterium-mediated T-DNA transformation or physical means such as protoplast transfection and microprojectile bombardment. These methods rely on mediators such as plasmids, ribonucleoproteins or viruses to carry the sequences to be introduced.

Plant protoplasts can be obtained by digesting cell walls with enzymes and editing reagents, that can be delivered by electroporation or by polyethylene glycol (PEG) treatment. Transfection of CRISPR/Cas components into protoplasts with subsequent regeneration of plants allowed to successfully introduce mutations with editing efficiencies ranging from 3% to 46%, resulting in either stable or transient expression in several plants including rice, soybean, *A. thaliana*, potato, grapevine, wheat, and lettuce [74,75,76,77,78,79,80,81]. This method allowed the creation of DNA-free edited plants by delivering preassembled Cas9-sgRNA ribonucleoproteins (RNPs) [79,80,82], which cannot be delivered by Agrobacterium [83]. The delivery of Cas9-sgRNA RNPs instead of plasmids that encode Cas9-sgRNA avoids that plasmids are degraded in cells by nucleases, resulting in small DNA fragments that may undesirably be inserted in the host genome [84]. This method has the ability to deliver multiple components to a large number of transfectable cells and to obtain vector less or DNA-free plants, since regenerants are obtained from single genetically modified protoplasts. This is an important advantage as plants edited using transfection of protoplasts may not be subjected to the regulatory issues and ethical barriers associated to transgenic plants. However, if this technique is used for knock in, an exogenous DNA template is required and regulation may no longer be avoided. In addition, protoplast transfection is in many cases associated with problems with plant regeneration and presence of undesired somaclonal mutations.

Another method used to deliver CRISPR/Cas components in plants is biolistic bombardment. It consists of coating microprojectiles—generally gold, silver, or tungsten particles—with DNA constructions which are then fired into plant cells with high pressure to penetrate the cell wall. Biolistic bombardment has introduced targeted mutations into plants, by using gold particles to carry and deliver CRISPR/Cas9 reagents in plasmids, causing stable integration in rice, wheat and soybean genomes, with editing efficiencies ranging from 14.5% to 76% [31,85,86]. Other study achieved TECCDNA (transiently expressing CRISPR/Cas 9 DNA) in wheat with editing efficiency of 1–9.5% [35]. Edited plants, without alien DNA integration, were obtained by biolistic delivery of RNP in maize [87] and wheat [88] with editing efficiencies that range from 21.8% to 47%. A geminivirus *Wheat dwarf virus*-based vector, pWDV2, carrying both Cas9 and sgRNA was used for biolistic transformation in wheat, providing a 12-fold increase editing efficiency when compared to the delivery of this system by traditional vectors [81]. The use of viruses to deliver CRISPR/Cas components will be further discussed in this review. Biolistic bombardment is usually efficient, multiple constructs can be delivered simultaneously and it can be used for many plant species. The major disadvantage is that it leads to multiple copies of the introduced genes, with random integration within genomes, which can lead to phenomena such as gene suppression in the recovered transgenic plants. It is also more costly than other methods.

To date, the most common system used to obtain transgenic plants is based on *Agrobacterium tumefaciens*. This approach has been widely used to deliver CRISPR/Cas components into plant cells of a variety of plant species. Agrobacterium has the ability to transfer a piece of its genome (T-DNA) to the cell nucleus, where it randomly integrates the plant genome [89]. Cas9 and sgRNA expression cassettes can be easily cloned into Ti plasmid, transformed into Agrobacterium and then introduced into plants. Many studies have used *A. tumefaciens* to deliver CRISPR/Cas components into plant cells, providing the insertions of T-DNA and achieved stable integration of transgenes in the genomes of many plant species—such as sorghum, *A. thaliana*, rice, tomato, maize, grapevine, aspen, rapeseed, and watermelon—with editing efficiencies that ranged from 23% to 100% [36,75,90,91,92,93,94].

Agrobacterium may also be used for transient expression of Cas9/sgRNA (agroinfiltration) [95]. This has been achieved in citrus with editing efficiency of 20% [33]. In *N. benthamiana*, rice and *A. thaliana*, viral transient expression resulted in editing efficiencies reaching 85% [23,62,96]. The use of viruses to deliver CRISPR/Cas components will be further discussed in the following section.

*Agrobacterium rhizogenes* has also been used for genome editing, resulting in stable integration of foreign DNA in soybean and a few other plant species, with editing efficiencies that range from 14.7% to 95% [97,98,99]. *A. rhizogenes* indicates a successful editing event by the appearance of hairy roots, however it requires regeneration of whole plants from these roots, which can be problematic for some species.

Agrobacterium-mediated delivery presents several advantages, it requires technology available in most laboratories, it is cheap, it allows multiplex editing as multiple binary vectors can be delivered into Agrobacterium and co-transformed into plant cells. Additionally, it can be used in transient assays, which may result in a non-transgenic plant and in a lower number of edited off-target sites.

### The Use of Viruses to Carry CRISPR Components

Many viruses, including retroviruses, adenoviruses and adeno-associated virus, have already shown to achieve effective delivery of genome-engineering reagents in mammalian systems [100,101].

In plants, *Tobacco mosaic virus* (TMV) was the first virus to be manipulated as vector, resulting in virus-induced gene silencing (VIGS) of an endogenous gene in *N. benthamiana* [102]. Since then, many other viruses have been widely used as vectors of gene silencing and for expression of foreign proteins in plants. However, their specific use to deliver genetic material such as CRISPR/Cas components in plants is much more recent. The first reports of the use of viruses to assist CRISPR/Cas gene editing, were in 2014 and were based on geminiviruses [103]. Since then, studies have been focused not only on the use of the DNA geminiviruses [23,81,96,104,105] but also on RNA viruses [40,62,106,107,108,109,110,111] as sgRNA delivery systems.

The numerous studies on the use of geminiviruses as vectors, result mostly from their easy manipulation. Geminiviruses (family *Geminiviridae*) are widespread, insect-transmitted and infect a wide range of plants [60,112]. Geminiviruses have a single stranded circular DNA with monopartite or bipartite genomes that range between 2.5 kb to 3 kb, with four to six open reading frames (ORFs). Once inside a plant cell, their single stranded genome forms a double stranded intermediate which is then used as template for transcription and for rolling-circle replication. They require only one replication initiator protein, Rep (C1), to initiate rolling-circle replication inside the host. Following replication, single stranded genomes are either converted to double stranded intermediates to initiate another replication cycle, or encapsidated by the coat protein to produce virions which then move to adjacent cells through plasmodesmata. Their small sizes mean they are easy to manipulate but on the other hand, it physically limits their cargo capacity; as so, they are unable to carry long DNA fragments, such as genes encoding Cas nucleases (~4.2 kb) [113].

To retain most of the features required for movement and replication, the CP of some bipartite begomoviruses may be replaced by the desired heterologous sequence of up to 800 bp or up to 1000 bp with further modifications [96,103,114]. However, with this change, geminiviruses are still unable to carry long DNA fragments such as genes encoding Cas nucleases, but it is enough to express and produce high amounts of sgRNA. In fact, the number of double stranded intermediates during viral replication is higher in the absence of the CP, possibly because the CP sequesters and packages ssDNA to form viral particles.

To increase cargo capacity, geminiviruses have been manipulated into non-infectious replicons (GVRs) by removing movement protein (MP) and coat protein (CP) coding sequences, and thereby eliminating cell to cell movement and insect transmission. In these cases, viral vectors are not infectious on their own and must be delivered into plant cells using Agrobacterium mediated transformation, in contrast to the possibility of agroinfiltration or mechanical inoculation for virus-induced gene editing (VIGE). These deconstructed DNA replicons have been used to introduce large amounts of repair templates in plants, which are required for HDR to outcompete NHEJ, showing high efficiency of HDR in plants.

Several studies have shown the use of geminiviruses to assist CRISPR/Cas (Table 2). Baltes et al. [103] used *Bean yellow dwarf virus* (BeYDV) replicons to efficiently deliver a sequence-specific nuclease (Cas9) and a repair template to tobacco plants for gene targeting, showing a considerable cargo capacity and with gene targeting frequencies with two orders of magnitude increase over conventional Agrobacterium T-DNA transformation. The use of BeYDV replicons also allowed genome editing in potato, by causing mutations capable of supporting a reduced herbicide susceptibility phenotype, while Agrobacterium T-DNA transformation held no detectable mutations for the same phenotype [104]. Cermark et al. [105] used BeYDV replicons to insert a strong promotor upstream of a tomato (*Solanum lycopersicum*) gene that regulates anthocyanin synthesis (ANT1) and obtained efficiencies 12-fold higher than traditional Agrobacterium T-DNA delivery. Similar efficiencies were obtained by Yin et al. [96] who used *Cabbage leaf curl virus* (CaLCuv) for VIGE by replacing viral CP by sgRNA, to edit different genes (NbPDS3 and NblspH) in *N. benthamiana* plants. VIGE makes use of Cas9 overexpression in plants and transient delivery of geminivirus vectors carrying sgRNAs and can be used as an alternative to VIGS.

In 2017, *Wheat dwarf virus* (WDV) replicons were used for gene targeting in wheat and rice [23,81]. WDV replicons showed high gene targeting efficiency and allowed to target multiple genes within the same cell [81]. Using this WDV-based system, Wang et al. [23] showed efficient HDR in rice.

In addition to geminiviruses, many RNA viruses have been used as vectors in plants (Table 2).

RNA virus-based vectors have the advantage of not integrating plant genome accidentally, so resulting in DNA-free plants, which avoids raising additional regulatory and ethical issues.

One of such virus-based vector, also widely used for VIGS, is *Tobacco rattle virus* (TRV) [115]. TRV belongs to genus *Tobravirus*, family *Virgaviridae*; it infects over 400 plant species and is transmitted by nematodes of the family *Trichodoridae*. It has a bipartite genome with two positive sense single stranded RNAs, RNA1 (TRV1), and RNA2 (TRV2). TRV1 is essential for virus replication and movement and TRV2 genome has genes encoding the CP and nonstructural proteins involved in nematode transmission. For its use as vector, these non-structural proteins in TRV2 can be replaced for the fragments of interest [116].

The first application of TRV as vector for genome engineering was in a non-transgenic approach for zinc-finger nucleases (ZFN) delivery in plants, by replacing RNA2 with RNA for the Zif268: FokI ZFN. In this system, targeted genome modifications were recovered at an integrated reporter gene in somatic tobacco and petunia cells, and transmission of mutations to next generation confirmed the stability of the ZFN induced changes [117].

The first use of TRV as a vector for CRISPR was in 2015, when TRV was developed as a vehicle for delivery of sgRNAs to modify genomes of *N. benthamiana* and *A. thaliana* [115]. A TRV vector containing sgRNA for phytoene desaturase gene (PDS) was introduced into leaves of *N. benthamiana* transgenic lines overexpressing Cas9, via agroinfection, which showed modification of the PDS gene [115]. In addition, TRV showed the ability to infect germline cells, as TRV-mediated delivery of sgRNA was not limited to infiltrated plants, allowing to successfully recover the desired modification in the next generation [115]. TRV can carry DNA fragments up to 3000 bp, however it is still not enough for the Cas gene, having been used only for sgRNA delivery into transgenic plants stably expressing Cas nuclease, thereby requiring that all genome edited plants are transgenic.

TMV, as mentioned previously, was the first virus to be manipulated as vector in plants, and has shown high level of accumulation and gene expression in several hosts, as well as prolonged integrity of its derived gene vectors [107,118]. Based on this potential, TMV was also developed as a vehicle for delivering sgRNA by partially substituting the CP with a sgRNA [107]. TMV showed to mediate target gene editing by showing the ability to deliver high concentrations of sgRNA and to efficient edit the target host gene in *N. benthamiana* plants, that was previously infiltrated with a plasmid expressing Cas9 [107].

Ali et al. [106] demonstrated that *Pea early browning virus* (PEBV) was able to deliver sgRNAs, resulting in mutagenesis of the targeted genomic loci in *N. benthamiana* plants, constitutively overexpressing the Cas9, in a more efficient way than TRV. In addition, like TRV, PEBV can infect meristematic tissues [119] which may allow the recovery of seeds with the desired mutations and obviate the need for tissue culture to generate heritable targeted mutations. *Barley stripe mosaic virus* (BSMV) has also been engineered as a sgRNA delivery system for CRISPR/Cas9 mediated targeted mutagenesis in wheat and maize, both transformed constitutively with Cas9 [108]. Recently, *Beet necrotic yellow vein virus* (BNYVV)-based vectors were designed to allow simultaneous expression of multiple foreign proteins and used for efficient sgRNA delivery for genome editing in transgenic *N. benthamiana* plants expressing Cas9 [109].

*Foxtail mosaic virus* (FoMV) has also showed to express sgRNAs in *N. benthamiana*, *Setaria viridis* and maize plants constitutively expressing Cas9, demonstrating that FoMV can enable gene editing [110].

All these previous attempts using plant RNA viruses for expression of sgRNA were able to express sgRNAs and introduce mutations into plant genomes that were overexpressing Cas9.

Until recently, there were no reports of delivery of the entire CRISPR/Cas system into plants through viral vectors due to their small capacity for carrying DNA/RNA fragments [120]. This was overcome when technical breakthroughs in delivering all CRISPR/Cas components into plant cells using negative-strand viruses were reported [121,122]. The negative-strand viruses, *Barley yellow striate mosaic virus* (BYSMV) and *Sonchus yellow net rhabdovirus* (SYNV), were used to successfully deliver CRISPR/Cas reagents and sgRNAs into plant cells. Ma et al. [122] showed that SYNV was able to knock out different genes in plants, achieving highly efficient DNA-free genome editing. This study also showed the multiplex editing ability of virus-delivered CRISPR/Cas9 system by designing sgRNAs for different genes without affecting the efficiency, and confirmed that genome-edited plants pass the genome alteration to subsequent generations. However, rhabdoviruses rarely infect germline cells, and SYNV mediated genome editing only works efficiently in somatic cells being plant tissue culture required to obtain an individual genome edited plant.

More recently, *Potato virus X* (PVX) has also been used to efficiently deliver both Cas9 and sgRNA into *N. benthamiana* plants [111]. PVX has a filamentous flexible structure with a 6345 nt (+) ssRNA, and each particle contains ~1350 coat protein subunits [123]. In opposition to what happens to small viruses, it is not likely that gene insert size is physically limited in PVX. Cas9 and sgRNA were placed between Triple Gene Block (movement proteins MP1, MP2, and MP3) and the CP of PVX and virus vector was both agroinfiltrated and mechanically inoculated in *N. benthamiana* plants. PVX-Cas9 RNA showed to infect most cells and express a large amount of Cas9 protein, while T-DNA integration into *N. benthamiana* genome occurred at low frequency. In addition, the mutation introduced was inherited by the next generation, but no PVX RNA was detected in these plants, showing that PVX was not transmitted through seed, leading to the suggestion that transgenerational transmission of PVX is unlikely to occur, resulting in DNA-free genome edited plants [111]. The possibility of such as simple and efficient virus-vector mediated delivery as the mechanical inoculation of a virus carrying the entire CRISPR/Cas system greatly facilitates transgene free gene editing in plants.

## 5. Challenges in the Use of Viruses for CRISPR

Virus mediated delivery of CRISPR/Cas is an easy way to deliver Cas nuclease and sgRNAs into plants, that overcome many challenges of transgene delivery, with no additional requirements, allowing to edit a desired feature into a plant, in laboratory or in the field, to obtain an improved DNA-free plant. They present several advantages such as they are easy to manipulate; viral genome can be used as repair template; they replicate to high copy number and accumulate at high levels (including sgRNAs and repair template) and systemically spread in a large number of plants leading high level expression and genome editing efficiency; multiple sgRNAs can be expressed from a single viral genome, allowing multi targeted genome editing; VIGE phenotypic alterations appear in plants in a relatively short time. In fact, VIGE is a promising tool for transgene integration-free genome editing, as it may not require the production of transgenic lines or simplify this operation, which is often laborious and time consuming, expensive, and raises public concerns and extra regulations [124,125].

In addition, some viruses have shown the capacity of invading meristems when used as CRISPR/Cas vectors, by systemically deliver sgRNAs and therefore enabling the recovery of progeny carrying the targeted genomic modification, overcoming the need of tissue culture—i.e., start from leaf tissue and regenerate the whole plant and then genotype for the presence of the modification [62,106], and opens new possibilities for producing plants with desired characteristics without the need of laborious and time consuming steps. Therefore, as a vector for genome engineering, it is highly desirable that viral vector infects germline cells, so that it will be possible to harvest mutant seeds from infected plants.

VIGE, especially RNA-based, may also contribute to decrease off-target activities, a major issue in CRISPR that occurs due to sgRNA mismatches and continuous expression of Cas nucleases, that result of editing unintended sites in the genome [126]. When viruses are used to express CRISPR/Cas systems, these will only be expressed when viruses invade plant cells, limiting the concentration of Cas and thus more likely that no off-target effect is detected [127].

Despite all these advantages, the limited cargo capacity that many viruses present (typically <1 kb) is a major drawback for their use for delivery of all gene editing reagents such as Cas9 (approx. 4.2 kb), as excess cargo results in the loss of systemic movement or loss of the cargo DNA [128].

For this reason, viruses have been developed to deliver sgRNAs to transgenic plants expressing Cas9 or have been deconstructed into non-infectious replicons or, more recently, a negative sense RNA virus and PVX showed to be able to carry the entire CRISPR/Cas system. All these studies show the huge possibilities and great potential of the use of plant viruses as vectors to efficiently target and deliver CRISPR/Cas reagents.

Further research may result in new discoveries that may allow positive-strand RNA or DNA viruses to be engineered to carry large DNA/RNA sequences without affecting their infectivity and with even greater editing efficiencies.

## 6. Concluding Remarks and Future Prospects for CRISPR in Agriculture

CRISPR/Cas technology has definitely simplified gene engineering showing great potential on improving several traits in plants, not only on the development of resistance to viral pathogens, but also to fungi, bacteria and insects, as well as tolerance to abiotic stresses and increase in yield [129,130,131] overcoming many difficulties of the techniques used until now [13,14,15]. This innovative technology at the disposal of plant breeding holds promise for protecting crops against abiotic and biotic stresses, so that farmers can meet consumers expectations for healthful and affordable products obtained by using few natural resources.

There are still technological improvements needed, such as precise editing and strategies to bypass the need for tissue culture. When using genome editing strategies, the possibility of editing unintended sites in the genome, off-targets, can never be ignored. As mentioned before, viruses as vectors of CRISPR systems may be used to decrease these collateral effects. In addition, a CRISPR/Cas technology in which a single nucleotide is chemically modified instead of producing DSB may also be widely used to prevent off-target effects [26].

Another constraint of the implementation of CRISPR as a plant breeding technique, is the difficulty to obtain new edited plants without tissue culture. Regeneration of plants through tissue culture is a time-consuming process, and there is the possibility of producing random somatic mutations. In addition, some crops are recalcitrant to regeneration through tissue culture. Delivery of CRISPR components in plant apical meristems so that seeds harvested will carry the mutations is desirable and already showed to be possible. However, many crop plants will lose valuable traits when propagated by seed.

Besides the technical and scientific aspects that must be overcome, CRISPR will also have to deal with social and political aspects such as the public concerns and government regulations mostly associated with transgenic plants. It is essential to provide clear information on CRISPR to the public and government to gain their acceptance and to influence regulatory policies on the use of CRISPR technologies in agriculture. The first clarification that must be done is that CRISPR may be applied to rapidly produce plants with traits that might easily also result from conventional plant breeding, as deletions and small insertions may also occur naturally or be induced during conventional plant breeding; or, in alternative, it can be used to introduce exogenous genes in plants and, only so, it would be equated with genetically modified organisms (GMO).

Plants subjected to CRISPR/Cas have gained extreme attention in terms of regulation. The United States Department of Agriculture (USDA) has recently regulated genome edited plants as safe for human consumption and the environment, as long as the resulting mutations are indistinguishable from mutations that occur naturally or by traditional breeding techniques. USDA has considered genome editing as an expansion of traditional plant breeding that can introduce new traits in plants more quickly and precisely, saving years or decades to bring needed new varieties to farmers, which is a great advance in the application of CRISPR in agriculture (Code of Federal Regulations, Vol. 7, part 340). This view has been adopted by most of the world, with the exception of the European Union, where, in 2018, the European Court of Justice (ECJ) ruled that genome edited organisms are GMOs until clarification of their legal status and, as so, are at present, subjected to the same obligations as transgenic organisms (Judgement in case C-528/16) and therefore fall under the European GMO Directive (2001/18/EC). The European Commission is currently carrying out a study on the potential of new genomic techniques that may play a role in sustainability, provided that resulting products they are safe for consumers and environment, as stated on the communication of ‘A Farm and Fork Strategy for a fair, healthy and environmentally-friendly food system’ (COM/2020/381), which is expected to be concluded in April 2021, and a different perception may be achieved. However, the current regulation is a clear obstacle to European agricultural innovation as greatly makes it difficult for genome-edited products to reach the market and has a huge impact in terms of competitivity with other countries with less restrictions.

CRISPR is a powerful plant breeding tool, which can contribute to provide food security to the ever-growing world population and to a sustainable agriculture, and discussions concerning the risks associated with genome editing should be driven more by scientific principles than by socio-political factors.

## Figures and Tables

**Table 1:** CRISPR/Cas for viral resistance in plants by targeting viral genome (virus mediated resistance) and host factors (plant mediated resistance).

Plant Species	Target Virus	Type of Resistance	Targeting Genomic Regions	Reference
*N. benthamiana*	BeYDV	DNA virus mediated	sgRNAs targeting LIR and rep/RepA	[63]
*A. thaliana* and *N. benthamiana*	BSCTV	DNA virus mediated	43 sgRNAs targeting BSCTV genome	[41]
*N. benthamiana*	TYLCV	DNA virus mediated	sgRNAs targeting Rep and CP	[62]
*N. benthamiana*	CLCuKoV	DNA virus mediated	sgRNAs targeting non-coding IR, CP and Rep	[40]
	TYLCV			
	TYLCSV			
	MeMV			
	BCTV-Worland			
	BCTV-Logan			
*A. thaliana*	TuMV	Plant mediated	elF(iso)4E knock out	[42]
*Cucumis sativus*	CVYV	Plant mediated	elF4E knock out	[49]
	ZYMV			
	PRSV			
*A. thaliana*	CYVV	Plant mediated	elF4E1	[51]
*Oryza sativa*	RTSV	Plant mediated	elF4G knock out	[50]
*A. thaliana*	CaMV	DNA virus mediated	sgRNAs targeting CP	[38]
*A. thaliana* and *N. benthamiana*	CMV	RNA virus mediated	22 sgRNAs targeting CMV genome and 3 sgRNAs targeting TMV genome	[13]
	TMV			
*N. benthamiana*	TuMV	RNA virus mediated	sgRNAs targeting HC-Pro and CP	[39]
Cassava	CBSV	Plant mediated	nCBP-1 and nCBP-2 (elF4E family)	[52]
Barley	WDV	DNA virus mediated	sgRNAs targeting MP, CP, Rep(Rep A and LIR	[64]
*N. benthamiana* and *Oryza sativa*	TMV	RNA virus mediated	sgRNAs targeting 5 regions in TMV, 3 in SRBDSV and 3 in RSMV	[15]
	SRBDSV			
	RSMV			
Banana *(Gonja manjaya)*	eBSV	DNA virus mediated	sgRNAs targeting ORF1, ORF2 and ORF3	[65]
Potato *(Solanum tuberosum)*	PVY	RNA virus mediated	sgRNAs targeting P3, CI, NIb and CP	[66]

**Table 2:** Viruses used to carry CRISPR sequences into plants and type of delivery.

Virus Type	Virus Family/ Genus	Virus Vector	Type of Delivery	Plant Species	Reference
ssDNA	*Geminiviridae/* *Mastrevirus*	BeYDV	Agrobacterium	*Nicotiana tabacum*	[103]
ssDNA	*Geminiviridae/* *Mastrevirus*	BeYDV	Agrobacterium	Potato *(Solanum tuberosum)*	[104]
ssDNA	*Geminiviridae/*	CaLCuV	Agrobacterium	*N. benthamiana*	[96]
	*Begomovirus*				
ssDNA	*Geminiviridae/* *Mastrevirus*	BeYDV	Agrobacterium	Tomato *(Solanum lycopersicum)*	[105]
ssDNA	*Geminiviridae/*	WDV	Protoplasts transfection	Wheat *(Triticum aestivum)*	[81]
	*Mastrevirus*				
ssDNA	*Geminiviridae/*	WDV	Agrobacterium	Rice *(O. sativa)*	[23]
	*Mastrevirus*				
+ ssRNA	*Virgaviridae/* *Tobravirus*	TRV	Agrobacterium	*N. benthamiana; A. thaliana*	[40,106,115]
+ ssRNA	*Virgaviridae/*	TMV	Agrobacterium	*N. benthamiana*	[107]
	*Tobamovirus*				
+ ssRNA	*Virgaviridae/* *Tobravirus*	PEBV	Agrobacterium	*N. benthamiana; A. thaliana*	[106]
+ ssRNA	*Virgaviridae/* *Hordeivirus*	BSMV	Agrobacterium	*N. benthamiana;* Wheat *(Triticum aestivum)*	[108]
+ ssRNA	*Benyviridae/*	BNYVV	Agrobacterium	*N. benthamiana*	[109]
	*Benyvirus*				
+ ssRNA	*Alphaflexiviridae/* *Potexvirus*	FoMV	Agrobacterium	Maize *(Zea mays),* Foxtail *(Setaria viridis), and N. benthamiana*	[110]
- ssRNA	*Rhabdoviridae/* *Cytorhabdovirus*	BYSMV	Agrobacterium	*N. benthamiana*	[121]
+ ssRNA	*Alphaflexiviridae/* *Potexvirus*	PVX	Agrobacterium Mechanical inoculation	*N. benthamiana*	[111]
- ssRNA	*Rhabdoviridae/* *Betanucleorhabdovirus*	SYNV	Agrobacterium	*N. benthamiana*	[122]
